# Cathepsin K knockout alleviates aging-induced cardiac dysfunction

**DOI:** 10.1111/acel.12276

**Published:** 2015-02-18

**Authors:** Yinan Hua, Timothy J Robinson, Yongtao Cao, Guo-Ping Shi, Jun Ren, Sreejayan Nair

**Affiliations:** 1 Division of Pharmaceutical Sciences & Center for Cardiovascular Research and Alternative Medicine, School of Pharmacy, College of Health SciencesLaramie, WY, 82071, USA; 2 WWAMI Medical Education, College of Health Sciences, University of WyomingLaramie, WY, 82071, USA; 3 Department of Mathematics, Indiana University of PennsylvaniaIndiana, PA, 15705, USA; 4 Department of Medicine, Brigham and Women's Hospital, Harvard Medical SchoolBoston, MA, 02115, USA

**Keywords:** aging, apoptosis, cardiac remodeling, cathepsin K, cardiac function, cardiac hypertrophy

## Abstract

Aging is a major risk factor for cardiovascular disease. It has previously been shown that protein levels of cathepsin K, a lysosomal cysteine protease, are elevated in the failing heart and that genetic ablation of cathepsin K protects against pressure overload-induced cardiac hypertrophy and contractile dysfunction. Here we test the hypothesis that cathepsin K knockout alleviates age-dependent decline in cardiac function. Cardiac geometry, contractile function, intracellular Ca^2+^ properties, and cardiomyocyte apoptosis were evaluated using echocardiography, fura-2 technique, immunohistochemistry, Western blot and TUNEL staining, respectively. Aged (24-month-old) mice exhibited significant cardiac remodeling (enlarged chamber size, wall thickness, myocyte cross-sectional area, and fibrosis), decreased cardiac contractility, prolonged relengthening along with compromised intracellular Ca^2+^ release compared to young (6-month-old) mice, which were attenuated in the cathepsin K knockout mice. Cellular markers of senescence, including cardiac lipofuscin, p21 and p16, were lower in the aged-cathepsin K knockout mice compared to their wild-type counterpart. Mechanistically, cathepsin K knockout mice attenuated an age-induced increase in cardiomyocyte apoptosis and nuclear translocation of mitochondrial apoptosis-inducing factor (AIF). In cultured H9c2 cells, doxorubicin stimulated premature senescence and apoptosis. Silencing of cathepsin K blocked the doxorubicin-induced translocation of AIF from the mitochondria to the nuclei. Collectively, these results suggest that cathepsin K knockout attenuates age-related decline in cardiac function via suppressing caspase-dependent and caspase-independent apoptosis.

## Introduction

Cardiovascular disease is the leading cause of mortality in the advanced world, and age is an important determinant of cardiac function. The morbidity and mortality rates associated with cardiovascular disease are significantly higher in the elderly than those in younger generations. Cardiac aging is a complex pathophysiological process accompanied by a number of biological events including cardiac remodeling and dysfunction (Lakatta, 1999; Boengler *et al*., 2009). Aging-associated cardiac abnormalities are manifested as diastolic cardiac dysfunction, cardiac hypertrophy, and fibrosis as well as impaired contractile function (Yang *et al*., 2005; Taneike *et al*., 2010; Hua *et al*., 2011). Although the precise mechanisms contributing to cardiac aging are far from clear, several postulates have been proposed. These include aging-induced mitochondrial damage, accumulation of reactive oxygen species, disruptions in intracellular Ca^2+^ homeostasis, and impaired excitation–contraction uncoupling (Yang *et al*., 2005, 2006). Recent studies have suggested an important role of both caspase-dependent and caspase-independent apoptosis in cardiac aging (Chiong *et al*., 2011).

Cathepsin K is a lysosomal cysteine protease that is highly expressed on osteoclasts and has been implicated in the pathogenesis of osteoporosis and other bone diseases (Yasuda *et al*., 2005; Lewiecki, 2009). Recent studies from our laboratory and those of others have shown that cathepsin K is expressed on cardiomyocytes (Cheng *et al*., 2012; Hua *et al*., 2013b) and this protease plays an important pathophysiological role in response to a variety of stressors including high-fat diet and pressure overload (Hua *et al*., 2013a,b). These studies demonstrate that ablation of the cathepsin K gene protects the heart from cardiometabolic stressors, suggesting that cathepsin K may serve as a potential target in the treatment or control of cardiac disease. At the molecular level, members of the cathepsin family have been shown to participate in apoptotic pathway in different cell types (Guicciardi *et al*., 2000, 2001). In response to stress, such as those induced by reactive oxygen species, the lysosomal membrane is disrupted and cathepsins leach out to the cytoplasm leading to cellular apoptosis (Ishisaka *et al*., 2001; Yuan *et al*., 2002). However, it is far from clear whether and how cathepsin K contributes to cardiac apoptosis in response to the triggers, especially those associated with cardiac aging. Here, we used a cathepsin K knockout mouse (*Ctsk*^*−/−*^) model to evaluate the myocardial geometry and functional properties in young and old mice, to test our hypothesis that cathepsin K ablation alleviates aging-induced cardiac dysfunction.

## Results

### Cathepsin K knockout reduces body weight, heart weight, and liver weight gain in old mice

As shown in Table1, body weight, heart weight, and liver weight were increased in wild-type (WT) old mice compared to young mice and these increases were attenuated in the cathepsin K knockout mice.

**Table 1:** General features of wild-type and *Ctsk*^−/−^ mice from young (6-month-old) and old (24-month-old) groups

Parameters	WT-young	WT-old	*Ctsk*^*−/−*^-young	*Ctsk*^*−/−*^-old
Body weight (g)	23.55 ± 0.68	27.1 ± 1.27*	24.39 ± 0.79	25.96 ± 0.57
Heart weight (g)	0.125 ± 0.007	0.182 ± 0.006*	0.134 ± 0.008	0.158 ± 0.006†^,^‡
Liver weight (g)	1.359 ± 0.051	1.602 ± 0.037*	1.557 ± 0.084	1.409 ± 0.080
Kidney weight (g)	0.279 ± 0.011	0.438 ± 0.020*	0.381 ± 0.026	0.423 ± 0.027
Spleen weight (g)	0.088 ± 0.006	0.11 ± 0.009*	0.099 ± 0.005	0.096 ± 0.015
HW/BW (mg g^−1^)	5.440 ± 0.146	6.741 ± 0.302*	5.512 ± 0.292	6.093 ± 0.211
LW/BW (mg g^−1^)	59.211 ± 0.656	59.378 ± 0.347	63.996 ± 3.340	54.646 ± 3.619

Values are mean ± SEM, *n* = 6–10 mice per group.

* *P* < 0.05 vs. WT-Young group.

† *P* < 0.05 vs. *Ctsk*^*−/−*^-Young group.

‡ *P* < 0.05 vs. WT-Old group.

### Cathepsin K knockout alleviates aging-associated impairment in cardiac performance

**Fig 1:**
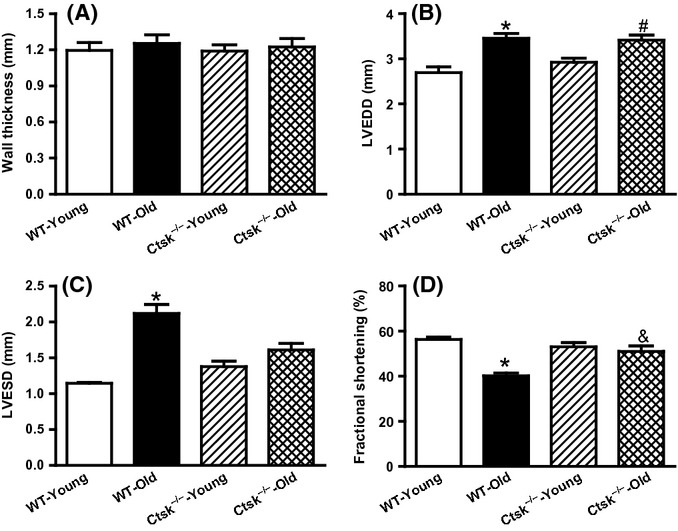
Echocardiographic features in young or old wild-type (WT) and cathepsin K knockout (*Ctsk*^*−/−*^) mice. (A) Left ventricular wall thickness. (B) Left ventricular end-diastolic dimension (LVEDD). (C) Left ventricular end-systolic dimension (LVESD). (D) Fractional shortening. Mean ± SEM, *n* = 5–9 mice group. *P < 0.05 vs. WT-Young group, ^#^*P* < 0.05 vs. *Ctsk*^*−/−*^-Young group, ^&^*P* < 0.05 vs. WT-Old group.

Results from echocardiographic analyses showed impaired cardiac performance in WT-old mice as evidenced by an increase in the left ventricular end-diastolic dimension (LVEDD), left ventricular end-systolic dimension (LVESD) as well as decreased fractional shortening (FS) (Fig.1B–D). Cathepsin K knockout reconciled the changes in the LVEDD and FS without significantly altering the LVESD (Fig.1B–D).

### Cathepsin K knockout attenuates aging-associated single cardiomyocyte contractile dysfunction

**Fig 2:**
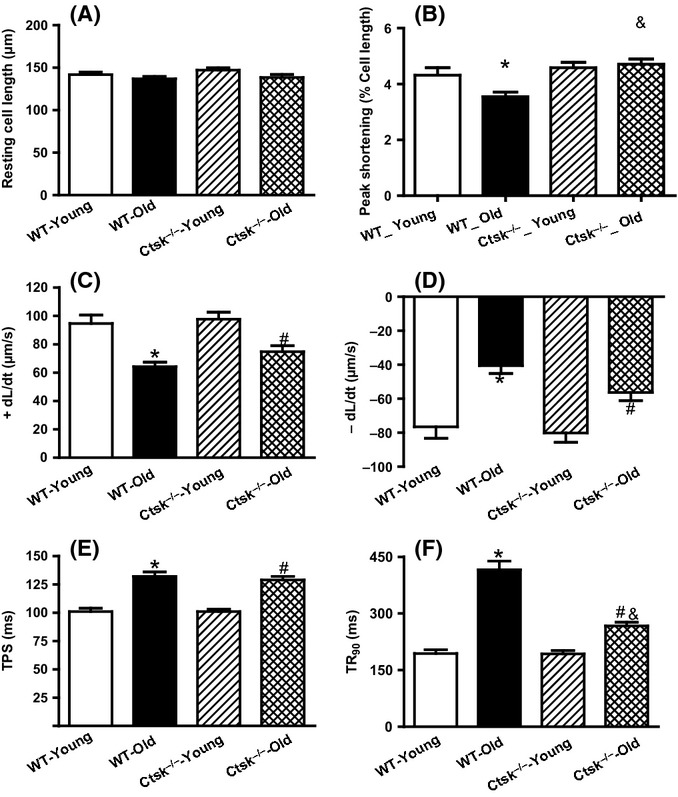
Cardiomyocyte contractile properties in young or old wild-type (WT) and cathepsin K knockout (*Ctsk*^*−/−*^) mice. (A) Resting cell length. (B) Peak shortening (normalized to cell length). (C) Maximal velocity of shortening (+dL/dt). (D) Maximal velocity of relengthening (− dL/dt). (E) Time-to-peak shortening (TPS). (F) Time-to-90% relengthening (TR_90_). Mean ± SEM, *n* = 87–108 cells per group, *P < 0.05 vs. WT-Young group, ^#^*P* < 0.05 vs. *Ctsk*^*−/−*^-Young group, ^&^*P* < 0.05 vs. WT-Old group.

To further investigate the effect of cathepsin K knockout on aging-associated myocardial dysfunction, contractile function of single cardiomyocytes was assessed. Although resting cell length was not affected by either age or genotype (Fig.2A), aging led to substantial impairment in peak shortening (PS), maximal velocity of shortening/relengthening (±dL/dt), time-to-peak shortening (TPS) as well as time-to-relengthening (TR_90_). Genetic ablation of cathepsin K genetic prevented the age-associated alterations of PS and TR_90_ (Fig.2B,F) without affecting ±dL/dt and TPS (Fig.2C–E).

### Cathepsin K knockout ameliorates aging-associated intracellular Ca^2+^ mishandling in single cardiomyocytes

As intracellular Ca^2+^ is the key ion responsible for contractile functions of heart, we sought to evaluate the cardiomyocyte Ca^2+^ handling in response to aging. As anticipated, aging was associated with a compromised intracellular Ca^2+^ handling by the cardiomyocytes (Supporting information, Fig. S1). This was evidenced by an elevation in the resting intracellular Ca^2+^ levels as well an increase in the intracellular Ca^2+^ decay rate. Interestingly, these changes were also observed in the aged-cathepsin K knockout mice (Fig. S1A, C and D). In contrast, however, cathepsin K knockout reconciled the age-associated reduction in intracellular Ca^2+^ release (Fig. S1B).

### Cathepsin K knockout inhibits age-associated cardiomyocyte hypertrophy, without altering fibrosis

**Fig 3:**
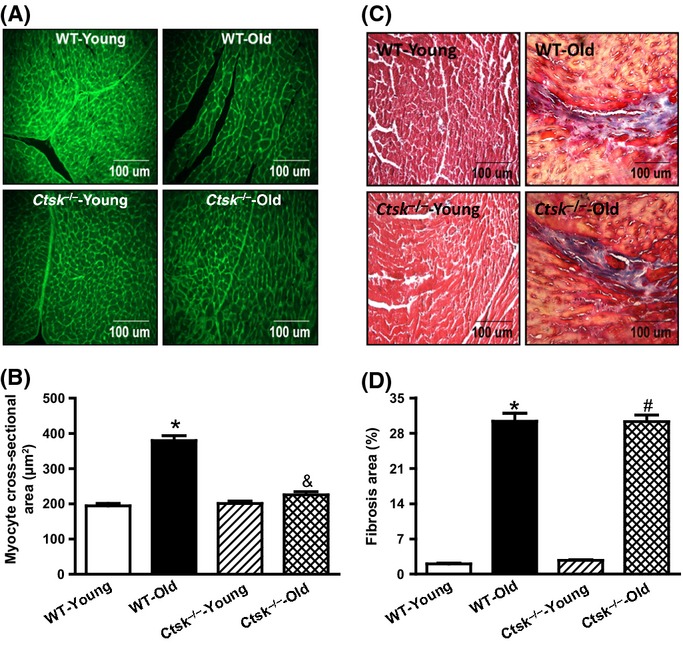
Morphologic changes in hearts from young or old wild-type (WT) and cathepsin K knockout (*Ctsk*^*−/−*^) mice. (A) Representative images using FITC-conjugated wheat germ agglutinin staining for cardiac tissues from young and old WT and *Ctsk*^*−/−*^ mice. (B) Quantitation of cardiomyocyte cross-sectional area. (C) Representative images of Masson's trichrome staining for cardiac tissues from young and old WT and *Ctsk*^*−/−*^ mice. (D) Quantitation of cardiac interstitial fibrosis. Mean ± SEM, *n* = 100 cardiomyocytes for cross-sectional area quantification, *n* = 20 sections for Masson's trichrome staining. *P < 0.05 vs. WT-Young group, ^#^*P* < 0.05 vs. *Ctsk*^*−/−*^-Young group, ^&^*P* < 0.05 vs. WT-Old group.

Consistent with the increased heart weight, the size of cardiomyocytes from WT mice increased in response to aging as evidenced by the wheat germ agglutinin (WGA) staining (Fig.3A,B). Interestingly, this aging-induced increase in cardiomyocyte size was not seen in the cathepsin K knockout mice (Fig.3A,B). Because cardiac aging is usually accompanied with increased fibrosis, we next assessed the extent of fibrosis in the hearts of these mice. As expected, the area of fibrosis was significantly elevated in the heart from WT-old mice compared to those of the WT-young mice. However, deletion of cathepsin K failed to inhibit the age-associated increase in cardiac fibrosis (Fig.3C,D).

### Cathepsin K knockout attenuates age-associated cardiac lipofuscin accumulation and expression of cell cycle decelerators

**Fig 4:**
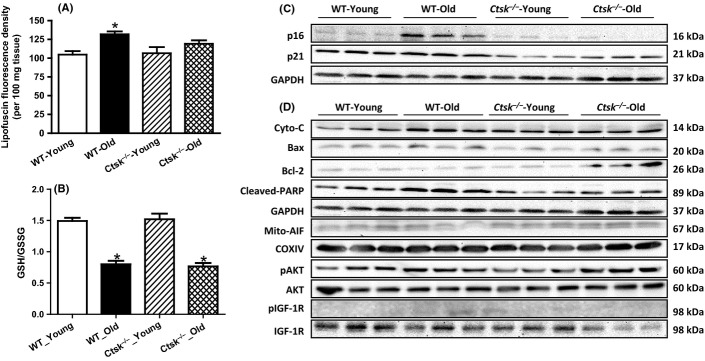
Effect of cathepsin K knockout on the contents of aging-related markers and activated cardiac apoptosis. (A) Cardiac lipofuscin content of young or old WT and *Ctsk*^*−/−*^ mice. (B) Cardiac oxidative levels measured by GSH/GSSG assay. (C) Representative Western blog image of cyclin-dependent kinase inhibitors p16, p21, and GAPDH (loading control). (D) Representative Western blot image of apoptosis-related proteins cytochrome *c*, Bax, Bcl-2, cleaved PARP, p-Akt, Akt, p-IGF-1R, IGF-1R, and GAPDH (loading control for above proteins), mitochondrial AIF and COXIV (loading control for mitochondrial AIF). Mean ± SEM, *n* = 4–6 hearts per group for lipofuscin test. *P < 0.05 vs. WT-Young group.

We next evaluated the accumulation of the lipofuscin pigments in the heart, the ‘tell-tale’ sign of aging. Our results demonstrate a robust upregulation of cardiac lipofuscin in WT-old mice, which was suppressed by cathepsin K knockout (Fig.4A). Furthermore, consistent with previous observations (Krishnamurthy *et al*., 2004; Naito *et al*., 2010), the expression levels of the cell cycle decelerator proteins p16 and p21 were elevated in the hearts of the WT-old mice, which was attenuated by cathepsin K knockout (Fig.4B and Fig. S2A,B).

### Cathepsin K knockout does not alter age-associated oxidative stress in the heart: elevated oxidative level in aged heart

Levels of cardiac oxidative stress were determined using glutathione (GSH)/glutathione disulfide (GSSG) assays. As shown in Fig.4B, age-associated decrease in the levels of GSH was unaltered by knockdown of cathepsin K suggesting that cathepsin K knockout may not attenuate oxidative stress.

### Cathepsin K deficiency inhibits age-associated basal phosphorylation of Akt and IGF-1

As insulin signaling pathway has been reported to be involved in the aging process, we next assessed the cardiac levels of phosphorylated Akt and IGF-1, the key mediators of the insulin signaling pathway. We noticed an elevation of phosphorylation of Akt and IGF-1 in response to aging, which was attenuated in the cathepsin K knockout mice (Fig. 4D).

### Cathepsin K deficiency blunts caspase-dependent and caspase-independent apoptosis in response to cardiac aging

**Fig 5:**
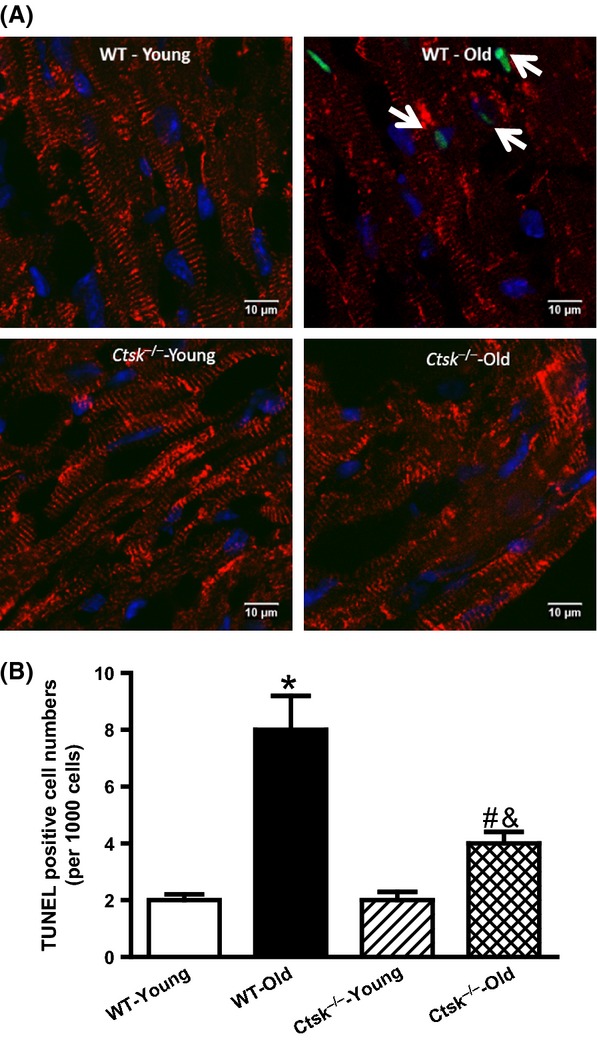
TUNEL staining for cardiac tissues from young or old WT and *Ctsk*^*−/−*^ mice. (A) Representative TUNEL staining of cardiomyocytes from heart sections of young or old WT and *Ctsk*^*−/−*^ mice. Apoptotic nuclei stained by TUNEL (green), counterstained with DAPI (blue) to label nuclei, and cardiomyocytes (desmin, red) were imaged by confocal microscopy. Arrowheads indicate apoptotic cardiomyocyte. (B) Quantitation of TUNEL-positive cardiomyocytes. Mean ± SEM, *n* = 1000 cardiomyocytes per group. *P < 0.05 vs. WT-Young group, ^#^*P* < 0.05 vs. *Ctsk*^*−/−*^-Young group, ^&^*P* < 0.05 vs. WT-Old group.

**Fig 6:**
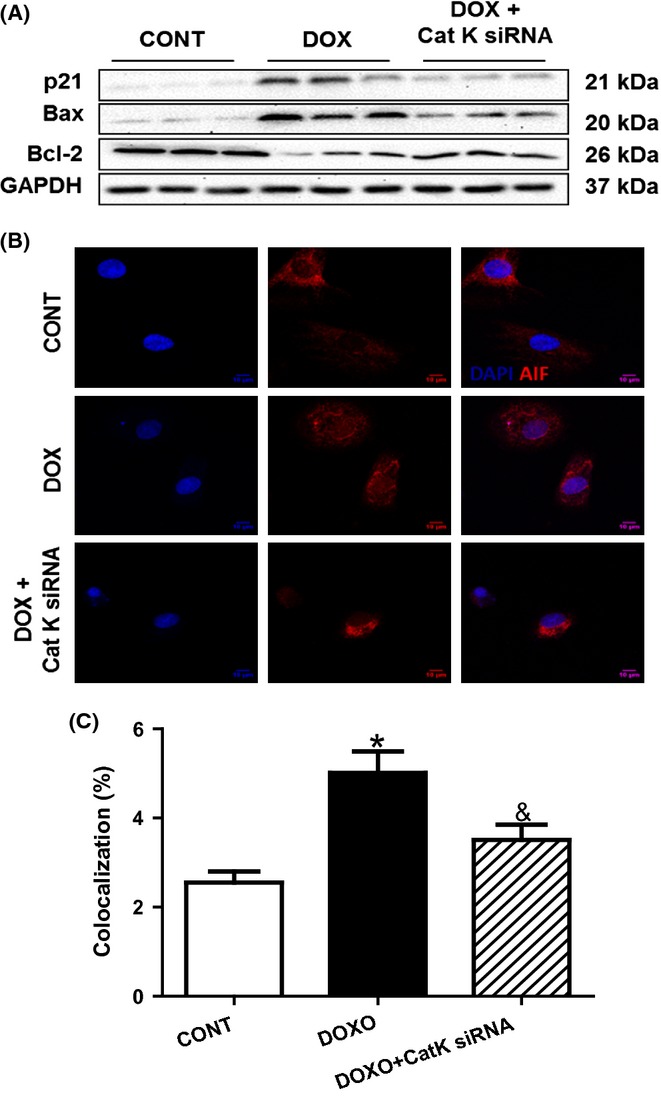
Effect of cathepsin K knockdown on doxorubicin-induced premature senescence, caspase-dependent and caspase-independent apoptosis in H9c2 cells. (A) Representative Western blot images of p21, Bax, Bcl-2, and GAPDH (loading control) of doxorubicin-challenged H9c2 cells in the presence or absence of cathepsin K knockdown. (B) Double staining for AIF and DAPI to indicate the translocation of AIF from mitochondrial to nuclei following doxorubicin treatment, which is rescued by cathepsin K deficiency. (C) Quantification of the overlap between two fluorescence dyes (red for AIF and blue for DAPI).

Expression levels of key proteins involved in the apoptotic cascade, including cytochrome *c*, BAX, and cleaved-nuclear enzyme poly (ADP-ribose) polymerase (PARP), were elevated in the hearts from the WT-old mice, but not in those of the cathepsin K knockout old mice (Fig.4C and Fig. S3). In contrast, we observed a reciprocal reduction in the protein levels of Bcl-2 (an anti-apoptotic protein) in the WT-old mice (Fig.4C and Fig. S3C) but not in the cathepsin K knockout mice. More importantly, the expression levels of the mitochondrial apoptosis-induced factor (AIF) were substantially decreased in the hearts of WT-old mice, suggesting the translocation of AIF from the mitochondria (Fig.4C and Fig. S3E) to the cytoplasm. In contrast, however, the AIF levels were restored in the mitochondria of the cathepsin K knockout mice. Furthermore, apoptosis, evaluated as TUNEL-positive cardiomyocytes, was elevated in the WT-aged heart and was attenuated by cathepsin K knockout (Fig.5A,B). In support of these observations, we found an increase in the expression levels of p16 and p21 in cultured cardiomyocytes (H9c2 cells) subjected to premature senescence by treatment with doxorubicin (Fig.6A and Fig. S4), which was attenuated by silencing of cathepsin K. Similarly, reciprocal changes in Bax and Bcl-2 proteins were also observed in cultured H9c2 cells upon treatment with doxorubicin, which was also countered by silencing cathepsin K (Fig.6A and Fig. S4). Additionally, the results from double staining of AIF and DAPI revealed the translocation of AIF from the mitochondria to the nuclei in response to doxorubicin, which was prevented by silencing cathepsin K (Fig.6A).

## Discussion

Compromised cardiac performance and suppressed capability to respond to stressors such as myocardial infarction in response to aging have been previously reported (Liu *et al*., 2012). In this study, we evaluated the effect of cathepsin K on aging-associated cardiac dysfunction and geometric alterations. The data from this study indicate that cathepsin K deficiency alleviates aging-associated cardiac contractile dysfunction, Ca^2+^ mishandling, and geometric anomalies. Additionally, age-associated cardiac hypertrophy was suppressed by cathepsin K knockout, without altering cardiac fibrosis. Age-associated increases in lipofuscin pigment, p16 and p21 proteins, and cardiac apoptosis were lower in the hearts of cathepsin K knockout mice compared to their WT counterparts. Consistent with these findings, elevated apoptosis was observed in H9c2 cells subjected to premature senescence by doxorubicin treatment. Furthermore, mitochondrial AIF levels were suppressed in aged hearts, and the nuclear translocation of AIF was suppressed in cultured cells following doxorubicin challenge. Both caspase-dependent apoptosis and caspase-independent cardiac apoptosis were rescued by ablation of cathepsin K both in aged mice and in senescent cells. We also observed that the knockout mice have lower weights, possibly indicating that they may be mildly calorically restricted throughout life. This could have also contributed to the apparent improvement in cardiac function. In summary, our data suggest that cathepsin K deficiency alleviates aging-associated cardiac dysfunction and geometric anomalies and that these effects are potentially regulated by the mitigation of aging-induced cardiac apoptosis.

Previous studies from our own group and others have documented an elevation of cathepsin K expression/activity in association with adipocyte differentiation (Xiao *et al*., 2006; Funicello *et al*., 2007), high-fat diet-induced obesity (Yang *et al*., 2008), obesity-associated cardiac dysfunction (Hua *et al*., 2013b), cardiac hypertrophy, and heart failure (Cheng *et al*., 2006; Hua *et al*., 2013a). To our knowledge, this is the first study to suggest a crucial role of cathepsin K in aging-associated cardiac anomalies. Although cathepsin K knockout reduced cardiac hypertrophy, it did not alter cardiac fibrosis associated with aging. This came as a surprise as we had expected to see changes in fibrosis associated with changes in cardiac mass. It is likely that cathepsin K has a nontraditional role in the heart, which may be different from its protease activity. Contributing to cardiomyocyte may represent one such nontraditional role of cathepsin K. Aging is usually accompanied with an increase in the generation of reactive oxygen species (ROS). In addition, aged mitochondria exhibit elevated activity of permeability transition pore (Mather & Rottenberg, 2000). The overproduction of ROS triggers apoptosis through damaged mitochondria. Under this condition, cytochrome *c* is released from mitochondria to initiate caspase-dependent apoptosis (Phaneuf & Leeuwenburgh, 2002). AIF, the major player for caspase-independent apoptosis, leads to DNA fragmentation and chromatin condensation when encountering specific stresses. AIF is normally localized in the mitochondria. However, when the mitochondria are damaged, AIF translocates to the nucleus to initiate apoptosis. It has been reported that inactivation of AIF inhibits apoptosis in embryonic stem cells (Joza *et al*., 2009). Interestingly, aging-associated accumulation of ROS has been shown to induce the translocation of AIF from mitochondria to nuclei and in turn lead to chromosome fragmentation (Susin *et al*., 1998). Furthermore, PARP regulates the translocation of AIF in cardiac myocytes subjected to oxidative stress (Chen *et al*., 2004). The inhibition of AIF translocation from mitochondria to nuclei in the cathepsin K knockout mice is attributable to its inhibition of PARP. Gore and colleagues had previously reported a causal involvement of cathepsin B in apoptosis in the liver cells (Guicciardi *et al*., 2000). Cathepsins have been shown to activate several key mediators of apoptosis including caspase-3 and Bid (Schotte *et al*., 1998; Ishisaka *et al*., 1999). Our study demonstrates the contribution of cathepsin K in aging-associated apoptosis, especially AIF-mediated caspase-independent apoptosis, further expanding the role of cathepsins in the process of apoptosis.

Collectively, our studies suggest that knockout of cathepsin K in mice and siRNA-mediated silencing of cathepsin K in cardiomyocytes prevent aging-associated impairment in cellular geometry and function. Our data suggest that the protective role of cathepsin K deficiency in cardiac aging is attributable to deactivating of aging-associated cardiac apoptosis, including caspase-dependent and caspase-independent apoptosis, and thereby protects against cardiac dysfunction (Fig. S5). Although the exact molecular mechanisms by which cathepsin K deletion protects hearts from aging need further study, our results provide a validation for the role of cathepsin K in age-associated cardiac anomalies. These observations are particularly significant given the fact that cathepsin K inhibitors that are currently under clinical trial for osteoporosis may have dual benefits of treating cardiovascular disease. This is an especially important result given our rapidly growing elderly population.

## Experimental procedure

### Experimental animals

The experimental protocols have been approved by the animal use and care committee at the University of Wyoming. 6-month-old (denoted as ‘young’) and 24-month-old (denoted as ‘old’) Cathepsin K global knockout mice (*Ctsk*^*−/−*^) and WT control (C57BL/6J) mice were used in this study. All mice were housed in a climate-controlled environment (22.8 ± 2.0 °C, 45–50% humidity) with a 12/12-light/dark cycle with free access to food and water.

### Echocardiographic assessment

Cardiac geometry and function were evaluated in anesthetized mice using a 2D guided M-mode echocardiography (Sonos 5500, Phillips Medical System, Andover, MA, USA) equipped with a 15- to 16-MHz linear transducer. FS was calculated from LVEDD and LVESD using the following equation:

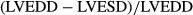
.

### Cardiomyocyte isolation and mechanics

Mouse cardiomyocytes were isolated using liberase enzymatic digestion; mechanical properties were assessed using an IonOptix™ soft-edge system (IonOptix, Milton, MA, USA) as described previously (Hua *et al*., 2011). Cell shortening and relengthening were assessed using PS, TPS, TR_90_ and ±dL/dt.

### Intracellular Ca^2+^ transients

A cohort of myocytes was loaded with fura-2/AM (0.5 μm) for 15 min, and fluorescence intensity was recorded with a dual-excitation fluorescence photomultiplier system (IonOptix). Cells were exposed to light emitted by a 75W lamp, while being stimulated to contract at a frequency of 0.5 Hz. Fluorescence emissions were detected between 480 and 520 nm; qualitative change in fura-2 fluorescence intensity (FFI) was inferred from the FFI ratio at the two wavelengths (360/380). Fluorescence decay rate was calculated as an indicator of intracellular Ca^2+^ clearing (Hua *et al*., 2011).

### Histopathological analysis

Ventricular tissues were stained with the FITC-conjugated WGA, (Sigma, St. Louis, MO, USA) and cardiomyocyte cross-sectional area was quantitated from 100 randomly selected cardiomyocytes. Myocardial fibrotic area was assessed using Masson's trichrome staining (Sigma).

### TUNEL staining

Apoptotic cardiomyocytes were detected using the In Situ Death Detection Kit (Roche, Branchburg, NJ, USA), with myocytes counterstained by Desmin antibody (Cell Signaling Technology, Beverly, MA, USA) and observed using a Zeiss confocal microscope. TUNEL-positive cells were quantitated by counting 1000 random cardiomyocytes.

### Determination of reduced and oxidized glutathione (GSH and GSSG)

The heart glutathione contents were measured as described previously (Guo *et al*., 2009). Tissue samples were sonicated in picric acid and centrifuged at 13 500 *g* for 20 min. The supernatant was then divided into two aliquots. One was used for total GSH assay and the other for GSSG assay. 100 μL of supernatant fractions with 2 μL vinyl pyridine was incubated at room temperature for 1 h to scavenge GSH for the GSSG determination. The GSSG was subtracted from the total glutathione to evaluate the GSH levels. GSH was determined by the DTNB-glutathione reductase recycling mechanism.

### Western blot analysis

Protein was extracted using a RIPA lysis buffer and Western blotted against antibodies for p16, p21 (Proteintech Group, Chicago, IL, USA), cytochrome *c*, Bax, Bcl-2, cleaved PARP, AIF, COXIV (used as the loading control for mitochondrial proteins), and GAPDH (loading control, Cell Signaling Technology). The signal was detected using a Bio-Rad Calibrated Densitometer (Hercules, CA, USA).

### Lipofuscin assay

Frozen heart tissues were homogenized in chloroform–methanol (1:20, w:v). The chloroform-rich layer was mixed with methanol following 15-min centrifugation at 15 000 *g*. Fluorescence in the sample was measured at an excitation wavelength of 350 nm and emission wavelength of 485 nm using a spectrofluorometer (Molecular Devices, SpectraMAX Gemini XS, CA, USA) (Shinmura *et al*., 2011). The data were expressed as fluorescence intensity per 100 mg tissue.

### Cell culture and RNA silencing

H9c2 myoblasts were cultured in a DEME medium and grown to 80% confluence. Cells were treated with doxorubicin (0.1 μm, 48 h) to induce premature senescence (Spallarossa *et al*., 2009). Cathepsin K protein deficiency was achieved by RNA silencing technique. In brief, small interfering RNAs against cathepsin K or control nontarget siRNA were transfected using DharmaFECT® transfection reagent (GE Healthcare, Lafayette, CO, USA) per manufacturer's instructions.

### Immunostaining for AIF in H9c2 cells

Double staining for AIF and DAPI was employed to indicate the location of AIF. In brief, cells were fixed in 4% paraformaldehyde for 15 min, followed by permeabilization with 0.2% Triton X-100 for 15 min. The cells were then blocked in 5% BSA for 30 min prior to incubation with an antibody against AIF at 4 °C overnight, followed by incubation in an anti-rabbit Alexa Fluor® 568 antibody (Life Technologies, Grand Island, NY, USA) at 37 °C for 60 min. The cells were counterstained by DAPI and viewed under a Zeiss confocal microscope.

### Statistical analysis

Data are presented as mean ± SEM (SEM = standard error of the mean). Statistical comparisons were performed using analysis of variance (anova) followed by Tukey's *post hoc* multiple comparison tests using the sigmaplot software (Jandel Scientific, San Rafael, CA, USA). Wherever indicated, permutation tests using the lmPerm package in the r statistical software (The R Foundation for Statistical Computing, Vienna, Austria) were performed to validate the statistical significance. The null hypothesis was rejected when *P* < 0.05.

## Funding info

This project was supported in part by grants from the National Center for Research Resources (P20RR016474) and the National Institute of General Medical Sciences (P20GM103432).

## Conflict of interest

The authors have no other conflict of interest to declare.

## Author contribution

YH – designed the experiments, conducted the study, and prepared the manuscript; GPS – reviewed the manuscript and contributed to the discussion; TJR – helped with the statistics and with reviewing the manuscript; YC – assisted with the statistical program; JR – reviewed the manuscript, contributed to the discussion, assisted with echocardiography and calcium measurements; SN – conceptualized the study, contributed to the discussion, and prepared the manuscript.

## Supporting Information

**Fig. S1** Intracellular Ca2+ transients in cardiomyocytes in young or old wild-type (WT) and cathepsin K knockout (*Ctsk*^−/−^) mice. **Fig. S2** Effect of cathepsin K knockout on cyclin-dependent kinase inhibitors in young vs. old mice. **Fig. S3** Effect of cathepsin K knockout on apoptotic markers in young vs. old mice. **Fig. S4** Effect of silencing of cathepsin K on doxorubicin (DOX)-induced premature senescence in cultured H9C2 cells. **Fig. S5** Schematic representing the role of cathepsin K in age-induced cardiac dysfunction.
