# Supplementary material for: Cathepsin K knockout alleviates aging-induced cardiac dysfunction
Source: Aging Cell. 2015 Feb 18;14(3):345–51. doi: 10.1111/acel.12276 (PMC4406663; doi:10.1111/acel.12276)
Supplement: Supplementary file 1 [file acel0014-0345-sd1.docx]

**Supplementary Figure 1.** Intracellular Ca2+ transients in cardiomyocytes in young or old wild-type (WT) and cathepsin K knockout (Ctsk-/-) mice. A: Resting fura-2 fluorescence intensity (FFI). B: electrically-stimulated rise in FFI (ΔFFI). C: single exponential intracellular Ca2+ decay rate. D: double exponential intracellular Ca2+ decay rate. Mean ± SEM, n = 99-110 cells per group. *p < 0.05 vs. WT-Young group, #p < 0.05 vs. Ctsk-/- -Young group, &p< 0.05 vs. WT-Old group.

**Supplementary Figure 2.** Effect of cathepsin K knockout on cyclin-dependent kinase inhibitors in young versus old mice. A and B: Densitometric quantitation of p16 and p21, respectively. Mean ± SEM, n = 3 hearts per group for Western blot assay, n = 4-6 hearts per group. *p < 0.05 vs. WT-Young group, &p< 0.05 vs. WT-Old group.

**F**


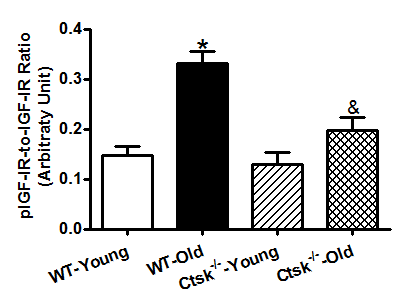


**G**

**Supplementary Figure 3.** Effect of cathepsin K knockout on apoptotic markers in young versus old mice. A-E: Densitometric quantitation of the Western Blots for cytochrome-c, Bax, Bcl-2, cleaved-PARP and mitochondrial AIF, respectively. Mean ± SEM, n = 3 hearts per group. *p < 0.05 vs. WT-Young group, ^&^p< 0.05 vs. WT-Old group.

**Supplementary Figure 4.** Effect of silencing of cathepsin K on doxorubicin (DOX)-induced premature senescence in cultured H9C2 cells. A-C: Densitometric quantitation of p21, Bax and Bcl-2, respectively. Mean ± SEM, n = 3. *p < 0.05 vs. control group, & p< 0.05 vs. DOX-treated group.

**
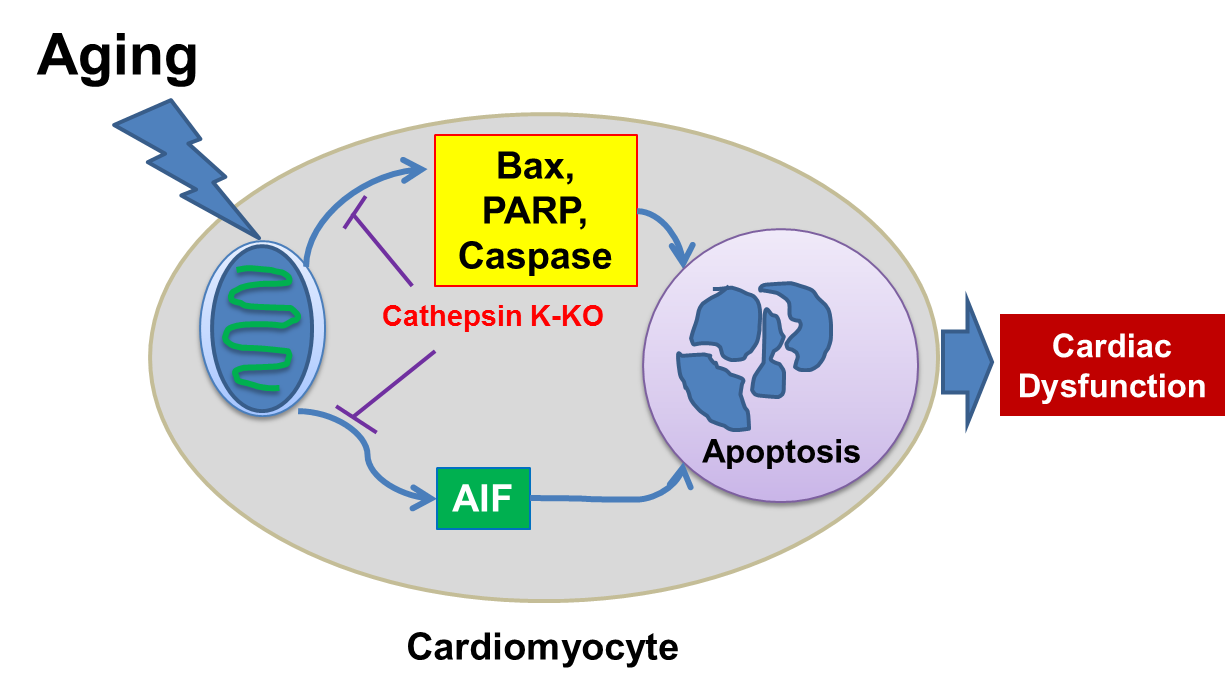
**

**Supplementary Figure 5.** Schematic representing the role of cathepsin K in age-induced cardiac dysfunction.
